# Quantifying neurologic disease using biosensor measurements in-clinic and in free-living settings in multiple sclerosis

**DOI:** 10.1038/s41746-019-0197-7

**Published:** 2019-12-11

**Authors:** Tanuja Chitnis, Bonnie I. Glanz, Cindy Gonzalez, Brian C. Healy, Taylor J. Saraceno, Neda Sattarnezhad, Camilo Diaz-Cruz, Mariann Polgar-Turcsanyi, Subhash Tummala, Rohit Bakshi, Vikram S. Bajaj, David Ben-Shimol, Nikhil Bikhchandani, Alexander W. Blocker, Joshua Burkart, Raphael Cendrillon, Michael P. Cusack, Emre Demiralp, Sarel Kobus Jooste, Alaa Kharbouch, Amy A. Lee, Joseph Lehár, Manway Liu, Swaminathan Mahadevan, Mark Murphy, Linda C. Norton, Tushar A. Parlikar, Anupam Pathak, Ali Shoeb, Erin Soderberg, Philip Stephens, Aaron H. Stoertz, Florence Thng, Kashyap Tumkur, Hongsheng Wang, Jane Rhodes, Richard A. Rudick, Richard M. Ransohoff, Glenn A. Phillips, Effie Bruzik, William J. Marks, Howard L. Weiner, Thomas M. Snyder

**Affiliations:** 1 Ann Romney Center for Neurologic Diseases and Partners Multiple Sclerosis Center, Brigham and Women’s Hospital, Department of Neurology, Harvard Medical School, Boston, MA USA; 2 Verily Life Sciences, South San Francisco, CA USA; 3 Biogen, Boston, MA USA

**Keywords:** Multiple sclerosis, Sensors and probes

## Abstract

Technological advances in passive digital phenotyping present the opportunity to quantify neurological diseases using new approaches that may complement clinical assessments. Here, we studied multiple sclerosis (MS) as a model neurological disease for investigating physiometric and environmental signals. The objective of this study was to assess the feasibility and correlation of wearable biosensors with traditional clinical measures of disability both in clinic and in free-living in MS patients. This is a single site observational cohort study conducted at an academic neurological center specializing in MS. A cohort of 25 MS patients with varying disability scores were recruited. Patients were monitored in clinic while wearing biosensors at nine body locations at three separate visits. Biosensor-derived features including aspects of gait (stance time, turn angle, mean turn velocity) and balance were collected, along with standardized disability scores assessed by a neurologist. Participants also wore up to three sensors on the wrist, ankle, and sternum for 8 weeks as they went about their daily lives. The primary outcomes were feasibility, adherence, as well as correlation of biosensor-derived metrics with traditional neurologist-assessed clinical measures of disability. We used machine-learning algorithms to extract multiple features of motion and dexterity and correlated these measures with more traditional measures of neurological disability, including the expanded disability status scale (EDSS) and the MS functional composite-4 (MSFC-4). In free-living, sleep measures were additionally collected. Twenty-three subjects completed the first two of three in-clinic study visits and the 8-week free-living biosensor period. Several biosensor-derived features significantly correlated with EDSS and MSFC-4 scores derived at visit two, including mobility stance time with MSFC-4 z-score (Spearman correlation −0.546; *p* = 0.0070), several aspects of turning including turn angle (0.437; *p* = 0.0372), and maximum angular velocity (0.653; *p* = 0.0007). Similar correlations were observed at subsequent clinic visits, and in the free-living setting. We also found other passively collected signals, including measures of sleep, that correlated with disease severity. These findings demonstrate the feasibility of applying passive biosensor measurement techniques to monitor disability in MS patients both in clinic and in the free-living setting.

## Introduction

Neurological diseases contribute to 2% of the global burden of diseases, and are increasing in prevalence.^1^ The accurate monitoring of neurological dysfunction for both clinical care and treatment trials remains a continued challenge, particularly given the availability of qualified neurologists and the complexity of assessment scales.

Multiple sclerosis (MS) is the most common cause of non-traumatic disability among young adults,^2,3^ and is classified as a demyelinating disease of the central nervous system. People with MS (PwMS) experience visual symptoms, gait difficulty, upper-limb weakness, spasticity, ataxia, fatigue, falls, disordered sleep, and autonomic dysfunction that can significantly impact quality of life.^3–5^ Symptoms and disability measures in PwMS are typically assessed at neurological visits every 3–12 months, with urgent visits for relapses. However, there may be significant fluctuation of symptoms, onset of relapses, and accrual of disability in between clinic visits that are not well measured by this clinical visit schedule.

Biosensors are non-invasive devices capable of measuring a variety of physiological and kinetic parameters such as overall activity, heart rate, body temperature, and other measures. Many biosensors are actigraphs and incorporate inertial measurement units (IMUs) to measure linear and angular forces.^6,7^ Such signals allow for building models to characterize a subject’s gait and other movement parameters. Some biosensors include optical sensors such as the photoplethysmogram (PPG) to allow for measurement of pulse oximetry and inference of heart rate. Wearable biosensors are increasingly being explored as measurement tools for disability in neurological diseases, including MS with the potential of delivering more quantifiable, objective, and meaningful measures of neurological function both within the clinic setting, and in between clinic visits.^8–15^

Most studies of wearable biosensors to date in PwMS have utilized commercially available products which focus on number of steps walked and total energy expenditure.^16^ These have consistently shown that patients with MS are less ambulatory in the community than controls. However, these sensors have limited ability to monitor more detailed metrics. One recent study compared several commercially available and research actigraphs, and found that commercially available monitors provided reasonably accurate estimates of total energy expenditure, but a larger error was noted for individual activities, particularly resistance exercise.^10^ A comparative study of smartphone accelerometer applications and motion sensors for capturing steps taken in PwMS found substantial variability in the precision and accuracy of the applications and devices.^17^

Here, we studied the use of research-grade biosensors in multiple body locations to quantify specific aspects of mobility like gait, balance, and dexterity in addition to more basic measures of activity both in clinic and during a free-living phase in a well-characterized cohort of PwMS. Our goals were to assess feasibility, adherence, as well as correlation of biosensor-derived metrics with traditional neurologist-assessed clinical measures of disability. We used algorithms to extract multiple features of motion and dexterity and correlated these measures with more traditional measures of neurological disability including the expanded disability status scale (EDSS)^18^ and the MS functional composite-4 (MSFC-4).^19,20^ We then explored the feasibility of measuring similar signals outside of the clinic by having subjects wear biosensors for 8 weeks while going about their daily lives. In order to contextualize this free-living data, we applied a deep neural network activity classifier to label different activities including walking segments and then used the same algorithms as in clinic to quantify motion and dexterity outside of the clinic.

## Results

### Cohort

The demographics and disability scores of the subjects at baseline visit are detailed in Supplementary Table 1. 92% (*N* = 23) of the cohort were females with 68% (*N* = 17) of the cohort being non-Hispanic women. The average age was 47 years old, with 16 years average disease duration at their baseline visit. The mean EDSS at baseline is 3.4, with a range of values from 1.0 to 6.5. The average EDSS scores were consistent across the three clinic visits. Two subjects experienced a relapse in the preceding 6 months from baseline visit and no relapses during the study period. There were no disease modifying medication changes during the study period.

### In-clinic assessments

#### Feasibility/adherence in clinic

Twenty-five subjects completed the initial clinic visit. 23 subjects completed the second clinic visit 4 months later as well as the 8-week free-living data collection. Twenty-one subjects completed the third clinic visit.

#### Biosensor correlations with in-clinic disease severity measures

We compared each of 23 features (detailed in the Supplementary Information) collected from the biosensor data and structured testing with in-clinic disease severity measures at each of the three clinic visits. Primary results for the EDSS, MSFC-4, and MSFC-4 composite for clinic visit 2 are shown in Table 1; additional results for clinic visits 1 and 3 are provided in Supplementary Tables 2 and 3.

**Table 1:** Spearman correlations of 23 in-clinic biosensor and structured testing measures with EDSS, MSFC-4, and MSFC-4 composite z-scores as observed at the second clinic visit (the first and third visits are summarized in the supplementary information).

Category	Feature	*N*	EDSS	MSFC-4 z-score	25 foot walk z-score	9-hole peg test z-score	Symbol-digit modality test z-score	LCVA test z-score
Mobility (gait)	Stance time	23	0.677**	−0.546**	−0.618**	−0.440*	−0.316	−0.447
	Swing time	23	0.469*	−0.425*	−0.484*	−0.380	−0.282	−0.451
	Mobility activity time	23	0.814**	−0.859**	−0.893**	−0.702**	−0.740**	−0.575
Mobility (turn)	Turn angle—chest	23	−0.444*	0.437*	0.442*	0.528*	0.400	0.230
	Turn duration—chest	23	0.021	0.125	0.150	0.286	0.148	0.026
	Turn velocity (max)—chest	23	−0.583*	0.653**	0.552*	0.597**	0.642**	0.456
	Turn velocity (mean)—chest	23	−0.563*	0.473*	0.382	0.436*	0.393	0.292
	Turn velocity (std)—chest	23	−0.588*	0.579**	0.476*	0.546**	0.618**	0.433
	Turn angle—ankle	23	−0.352	0.256	0.276	0.295	0.444*	−0.083
	Turn duration—ankle	23	0.004	−0.189	−0.140	−0.059	−0.028	−0.535
	Turn velocity (max)—ankle	23	−0.481*	0.594**	0.527*	0.628**	0.579**	0.463
	Turn velocity (mean)—ankle	23	−0.226	0.325	0.274	0.415*	0.371	0.236
	Turn velocity (std)—ankle	23	−0.520*	0.490*	0.533*	0.498*	0.501*	0.377
Mobility (balance)	Sway distance left–right	23	0.568*	−0.532**	−0.478*	−0.610**	−0.599**	−0.271
	Sway distance anterior–posterior	23	0.503*	−0.489*	−0.457*	−0.587**	−0.550**	−0.237
	Sway displacement left–right	23	0.513*	−0.518*	−0.378	−0.514*	−0.440*	−0.376
	Sway displacement anterior–posterior	23	0.184	−0.120	−0.021	−0.257	−0.110	−0.021
Overall activity	Heart rate variability	16	−0.519*	0.488*	0.434	0.391	0.677**	0.241
Fatigue	Mean PVT delay—Total	23	0.242	−0.486*	−0.510*	−0.422*	−0.542**	−0.429
	Mean PVT delay—1 challenge	23	0.360	−0.362	−0.347	−0.335	−0.291	−0.310
	Mean PVT delay—3 challenges	23	0.291	−0.456*	−0.489*	−0.432*	−0.360	−0.418
	Mean PVT delay—5 challenges	23	0.348	−0.508*	−0.542*	−0.489*	−0.431*	−0.469
	Mean PVT delay—7 challenges	23	0.304	−0.498*	−0.525*	−0.410	−0.508*	−0.412

*EDSS* expanded disability status scale, *MSFC-4* multiple sclerosis functional composite-4, *LCVA* low contrast visual acuity test

*p- and *q*-values 0.05, **p- and *q*-values 0.01

**Fig. 1:**
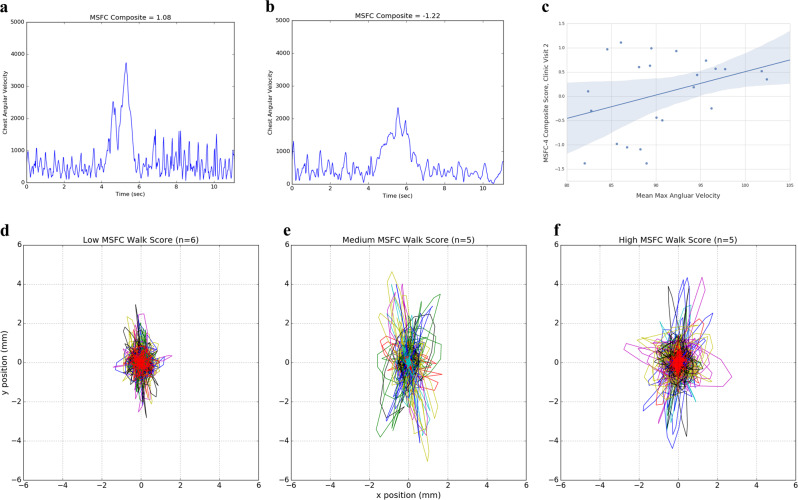
In-clinic measures correlated with disease severity at the second clinic visit. Gyroscopic measurements at chest during turns, measuring angular velocity, differs with disease severity. **a**, **b** Representative traces for turns from less (**a**) and more (**b**) disabled subjects for angular velocity during turns during the timed-up and go test. **c** Spearman correlation across the entire cohort for the mean max angular velocity of observed turns (95% confidence interval shown for trend line). Postural sway also shows increased deviation in both left–right (x) and anterior–posterior (y) directions as disability scores increase; individual traces (unique color by subject) for 30-s balance portions are shown for three cohort subgroups based on MSFC walk score for (**d**) low disability, (**e**) medium disability, and (**f**) high disability.

Several biosensor-derived features, including gait (stance time, turn angle, mean turn velocity) and balance, were significantly correlated with EDSS and MSFC-4 scores. Spearman correlation of mobility stance time with MSFC-4 z-score was −0.546 (*p* = 0.0070) at visit two. Significant correlations were observed for several aspects of turning, including turn angle (*r* = 0.437; *p* = 0.0372) and maximum angular velocity (*r* = 0.653; *p* = 0.0007) at visit two. Similar correlations were observed at other clinic visits. Figure 1a, b shows representative traces measured from the chest during the TUG test for two subjects with differing disability. Figure 1c shows the correlation across the cohort; slower turns are observed as MSFC-4 score decreases (more severe disability).

Balance also stood out as a significant feature in our analyses. Figure 1d shows body displacement for three severity groups based on MSFC walk score. Spearman correlations for both sway distance left–right (*r* = −0.532; *p* = 0.0090) and anterior–posterior (*r* = −0.489; *p* = 0.0179) to overall MSFC-4 z-score were significant. The overall magnitude of these changes is on the order of millimeters, making this a feature that may be difficult to observe by eye; thus use of a quantitative metric from a biosensor can complement clinical observation.

The Psychomotor Vigilance Task (PVT) also showed some correlations to the overall MSFC-4 score as well as several of the subscores. Overall, the in-clinic biosensor and structured activity features revealed multiple features across several domains of activity that merited further investigation outside of the clinic.

### Free-living assessments

#### Feasibility

Between clinic visits 2 and 3, 23 subjects wore a set of sensors while going about their daily lives. Roughly 50,000 h of data were collected across all the sensors during this period, with reasonable compliance (see Supplementary Fig. 1) throughout the 8 weeks with only a few exceptions, including participants traveling. Site coordinators did contact participants when data were not observed to help troubleshoot possible issues, which likely contributed to the favorable compliance observed in this study.

#### Associations of free-living measures with in-clinic disease severity measures

Given the unstructured nature of free-living data, use of an activity classifier was paramount to segment these data to identify walking and other activities; physiometric signals were then algorithmically extracted, as previously described. In addition to the clinically derived measures, measures related to activity, pulse rate and pulse rate variability, and sleep were added into the analysis at this stage. In total, 23 features were compared with the clinical scores captured at visit 2 before the start of this longitudinal data collection.

**Fig. 2:**
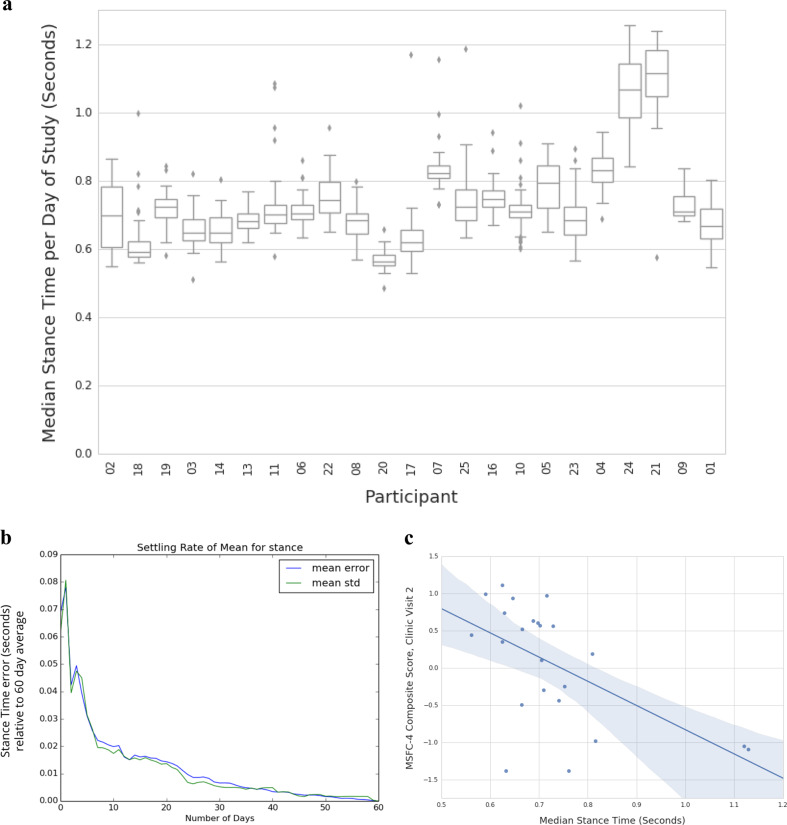
Properties of free-living mobility measures extracted during classified walking periods. **a** Box and whiskers plots representing the distribution of daily stance time measures for that subject across each of the 56 days of measurement during the study; subjects are sorted by MSFC-4 clinic visit 2 scores. **b** Variability of the Stance time median value by number of days of observation within subjects. A variable number of days (from 1 up to all 56 + days) was compared to the overall study time median, demonstrating how averaging different ranges of data can help control for the overall variability. With 1 week’s worth of data, the standard error for stance time is within 0.02 s compared to 0.08 s with just 1 day’s worth of data. **c** Spearman correlation for median stance time, calculated across the entire 8-week free-living period; there is a correlation (−0.56, *q*-value = 0.0052) between this free-living measure and the MSFC-4 composite score at visit 2.

Before completing the clinical comparisons, we analyzed the median values for these extracted data for each day of the study to understand the overall variability observed in a measure like stance time (Fig. 2a). Higher variability in the signal would be expected given the greater technical variability of data captured in this setting. In clinic, subjects are instructed to walk as fast as they can and to turn exactly 180° during a TUG test. In the real world, subjects may walk with variable stride rates and may turn in many different angles based on their walking paths. In our analysis, we found that aggregation over multiple days was necessary to remove the technical noise in the data. Comparing median stance time for a variable number of times to the overall median across 8 weeks of observation (Fig. 2b) shows that once one or more weeks are aggregated the mean errors of this measure drop to below 0.02 (compared to the overall study median for this measure). Figure 2c shows how the aggregated data over a 2-month window reveals statistically significant correlations to MSFC-4 measures captured in clinic.

For this initial feasibility study of free-living measures, we used the median values from all 2 months of free-living measurement to look for associations with clinically derived measures. Table 2 shows the seven most strongly correlated signals from this outside of clinic observation period.

**Table 2:** Associations of free-living features with MSFC span.

Category	Feature	Correlation	*p*-value, *q*-value
Mobility (gait)	Stance time	−0.56	0.0055, 0.0052
Mobility (turn)	Turn angle—chest	0.44	0.0377, 0.0154
Mobility (gait)	Swing time	−0.39	0.0648, 0.0215
Fatigue/sleep	Mean PVT Delay	−0.55	0.0060, 0.0055
Sleep	Movement rate of legs during sleep	−0.45	0.0398, 0.0154
Sleep	REM percent	0.42	0.0584, 0.0202
Overall activity	Idle minutes	−0.52	0.0110, 0.0074

Several of the mobility features observed in clinic to be significant, including stance time and turn angle (measured at the chest), replicated in this free-living setting without any structured activities to capture these features of motion. Consistent with earlier publications, overall activity levels were also correlated with disease status. Finally, several sleep and fatigue measures captured passively (REM percent/movement rate of legs) or actively (PVT testing) were significantly associated with MSFC-4 scores, with the best correlation with the SDMT measure of processing speed (Table 2). In contrast, patient-reported fatigue scores via daily questionnaire were not significantly associated with MSFC-4 scores.

#### Adherence to study protocol

During the free-living monitoring phase, we assessed compliance qualitatively based on both the number of hours that the wrist devices were worn per week compared to the protocol-defined target, and the consistency in the number of hours that devices were worn week to week across the 8 weeks of observation. We classified compliance to device-wearing into three behavioral groups. Two to three subjects were in a low compliance group with consistently low (<20% target) or no data return, particularly after the first couple weeks of the study. Three to four subjects were in a declining compliance group with initially high compliance but some decline week to week as the study went on; within this group, there was still data returned each week but by the end of the study it was at or below 40–60% of the study target in terms of number of hours. Eighteen subjects were in a high compliance group with consistently high data return, above 80% of the protocol-specific targets even if a couple weeks were at lower values. During the free-living monitoring phase, 83% of the hours of data were successfully received, while 17% of the hours of data expected was not received due either to non-adherence issues such as not wearing the biosensors or to technical issues.

#### Participant feedback

We solicited participant feedback at the conclusion of the study and 15/23 participants completed a survey. In response to the question: “How likely are you to recommend that a friend/colleague with MS participate in a similar study?” 87% responded with a score of 8 or greater on a scale with maximum of 10. In response to the question: “Any aspects of the study that have been difficult for you?”: 40% reported issues with either charging or synchronizing the device; 27% reported issues related to “ease of use”; 13% reported technical issues with the device; 7% reported no issues.

## Discussion

Our study found that research-grade wearable biosensors that capture accelerometry and gyroscopic motions provided metrics with good correlations to complex MS scales traditionally assessed by a neurologist. The biosensor measures most closely correlated with traditional neurological scales included those captured during passive walking, such as mobility stance, turn angle velocity, and postural sway. These measures could be reliably captured both in-clinic and during free-living activity, which opens the opportunity to use biosensors to measure and monitor neurological disability in between clinic visits. Additionally, the PVT activity correlated with MS disability scores including upper-limb subscores. Percent REM sleep, based on pulse rate variability captured by the device, correlated with disability scores. This study demonstrates the potential for biosensor-derived, passively collected measures for use in routine clinical monitoring of MS patients in the free-living setting, as well as for use in clinical trials to measure disability and relapses.

MS is an example of a complex neurological disease, which can affect a variety of neurological measures including gait, mobility, motor strength, vision, sensation and coordination of both upper and lower limbs. Though in-clinic measures continue to be useful for assessing disease progression, these only provide snapshots of an individual’s disease and may miss out on important variations that occur in between clinic visits.

Biosensors are emerging as important new means to capture neurological disability data, both in clinic and at home. Past studies in MS using wearable biosensors have relied on counting steps,^9–11,16^ which requires active participation by the subject, and may be influenced by a number of factors that enhance or preclude walking on a daily basis such as weather, workload, daily schedule and other non-biological factors. Passive measures, which do not require active participation, may thus enhance the ability to capture mobility and disability measures on a more routine basis, and may better reflect daily neurological function independent of volition.

Our findings identified several measures captured by 2–3 biosensors worn on a daily basis that provided good approximation to clinician-evaluated metrics. These included several walking measures captured by chest or ankle worn devices, deriving more specific aspects of gait including stance time and turn angle than previous studies focused on step counts. Future iterations of biosensor data will need to balance the wearability and convenience of devices with sensitivity to relevant signals. Studies such as this one will help to inform relevance.

MS patients also experience a variety of sleep disorders that can impact daily function.^21^ Disturbed sleep and abnormal sleep cycles are correlated with fatigue in MS patients.^22^ Sleep disturbances can be due to insomnia, as well as secondary sleep issues related to neurogenic bladder, pain or spasticity. Although several studies have investigated the use of actigraphy in patients with primary sleep disorders, Parkinson’s disease, psychiatric and other neurological disorders, few studies have examined the application to the study of sleep in MS. One study found sleep efficiency measured by actigraphy correlated with daily sleep ratings.^23^ In this study, we found that measurement of REM percent as well as movement of legs during sleep correlated with disability scores, indicating potential links between sleep quality and neurological dysfunction, which require further study to investigate cause versus effect relationships. In this study, REM% is estimated based on a heart rate variability model from the PPG signal, not gold-standard polysomnography. Further research is needed to confirm this result.

Our results therefore demonstrate the potential both in-clinic and outside of the clinic to assess a subject’s disease status. Limitations of this study included the fact that 21/25 subjects completed all study visits, and 15/23 completed the final survey. 18/25 subjects were classified as high compliance for the at-home portion of the study, which could be further optimized. Further analysis is underway to assess the day-to-day variability of biosensor metrics. Additional work is needed to understand exactly which features, measured in which body locations, are most reliably captured. Longitudinal data collection over many months or years may then allow for detection of changes in an individual’s disease status; the observation window in this study was too short to expect any such changes, and no clinically-determined relapses were measured during the study.

With further refinement these longitudinal data could be summarized for MS clinicians during the (semi-)annual neurologic exams to better contextualize the results of an EDSS/MSFC-4 assessment given (once or) twice a year. Further studies may also evaluate these measures in terms of treatment response, in particular for remyelinating and neuroprotective therapies, in which subtle disability changes may be challenging to detect during periodic clinic visits and with current disability measures.

These findings could be extrapolated to other neurological diseases, including Parkinson’s^24,25^ Alzheimer’s disease^26^ and stroke,^27^ in which free-living monitoring of disability accrual and response to treatment may be critical to tailoring care. Indeed, the first biosensor device to detect the occurrence of seizure was recently approved by the FDA, demonstrating the clinical utility of biosensor devices in neurological diseases.

The combination of wearable biosensors and machine-learning algorithms to annotate daily activities may advance the care of neurological diseases including MS by enabling the monitoring of patients in the free-living environment.

## Methods

### Subjects

Twenty-five MS patients followed at the Partners MS Center, Brigham and Women’s Hospital, were selected to comprise three severity cohorts based on EDSS scores in the prior 6 months (0–2.5, >2.5 to 4.0, and >4.0–6.5). Eight to ten subjects per EDSS grouping were enrolled. Subjects were selected from the ongoing prospective longitudinal study (CLIMB) at the center, in which detailed neurological examinations, EDSS scores, medication, and relapse history are collected in a relational database. Written informed consent was obtained from all subjects, and the study was performed in compliance with relevant guidelines and regulations Study subjects signed a written informed consent form and received study remuneration in the form of a gift card, as well as parking for the study visits. This study was approved by the Partners Healthcare Research Committee Institutional Review Board (IRB) and participants provided written informed consent to take part in the study.

### Biosensors

**Fig. 3:**
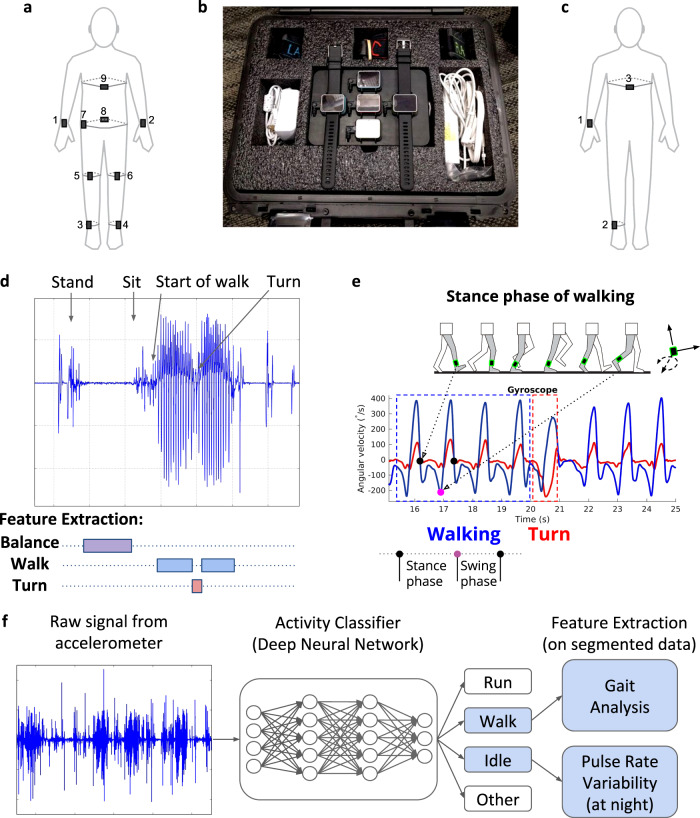
Experimental design and data segmentation. **a**–**c** Biosensor diagram, including (**a**) nine sensor locations used in clinic, (**b**) the free-living kit of biosensors for wrists, ankles, and chest given to participants and (**c**) locations for daily wear. **d** Example segmentation and featurization of data from the in clinic assessments, where an example trace from the left ankle accelerometer is shown during structured activities that included standing, maintain balance for 30 s, sitting, and then performing a 25-foot timed-up and go test with a 180 degree turn in the middle. **e** Example featurization (based on multiple angular velocity signals) for detecting stance and swing phase of a step when walking, as well as turns. **f** Workflow for classifying activity from unstructured free-living data, where an activity classifier takes raw accelerometer input from the wrist-worn biosensor to identify idle, walking, running, and other activity periods in the data. Segmented data for walking undergo gait analysis using same algorithms as used for in clinic data featurization and shown in (**e**). Idle minutes are used to assess pulse rate variability, particularly at night to estimate stages of sleep.

The study used the Cardiac and Activity Monitor (CAM), an investigational research device developed by Verily Life Sciences. The CAM device measured acceleration and motion with an on-board IMU, as well as heart rate by a PPG sensor or on-demand electrocardiogram (ECG) measurement. Additional sensors allowed measurement of skin impedance, body temperature, and environmental factors such as light exposure and air pressure. The CAM allowed for event tagging, patient-reported data collection, and other assessments by interaction with the screen. Although typically worn in a wrist form factor like a watch, we developed methods to reliably attach CAM to multiple body locations using different clips and straps (Fig. 3a–c).

In addition to the CAM devices, each subject received a laptop to perform additional testing as described below. Technical support was provided throughout the study through a call-in service which ensured high compliance by the subjects.

### In-clinic assessments

Subjects completed three clinical visits separated by approximately 16 weeks and 8 weeks, respectively. At each clinic visit subjects completed MSFC-4 (SDMT, T25FW, 9HPT, LCVA), and other testing while wearing biosensors at nine body locations. The calculation for the MSFC-4 and composite z-scores can be found in Supplementary Table 3. An EDSS assessment was performed by one neurologist at each clinic visit. Additional structured assessments were performed including:

- Standing still for 30 s to measure postural sway;
- Three 25 foot timed up and go tests (TUGs) and a 2 minute walk test to measure walking and turning;
- PVT test performed on the CAM to measure reaction time and assess fatigue.^28,29^

### Free-living assessments

In between the 2nd and 3rd clinic visits, subjects were given a set of biosensors for their wrist, ankle, and sternum which they wore for eight weeks while going about their daily lives. Measurements were captured throughout the study by daily syncing and upload of data when charging the devices. Most of these data were from unstructured, passive data collection. Subjects also were asked every day to complete a fatigue survey and to perform a PVT test. Subjects were requested to wear three biosensors (wrist, ankle and chest) during the day, and two biosensors at night (wrist and ankle only).

### Biosensor feature extraction for in-clinic data (a full listing of features are given in the Supplementary Information)

Algorithms were developed by Verily using videographic and biosensor-derived data from healthy controls to extract characteristics of walking (including steps taken, stance time and swing time of each limb), turning (angle, duration, and angular velocity of turns from chest or ankle body locations), and balance (sway distance and displacement in both left–right and anterior-posterior directions). These algorithms were applied to the structured in-clinic assessments with data segmentation (Fig. 3d, e). From the PVT tests, mean delay for the entire 3-minute test was calculated as well as the mean delay for the first 1, 3, 5, and 7 challenges during the PVT test.

### Biosensor feature extraction for free-living data (a full listing of features are given in the Supplementary Information)

Outside of the clinic, an additional step of activity classification was required to interpret the data signals (Fig. 3f). A deep neural network was trained on tagged activity data from healthy adults to classify walking, running, typing/hand movement, and other activities. Walking minutes identified by this activity classifier were analyzed by the same algorithms as in-clinic data to extract motion features. In addition to signals of mobility and gait assessed in clinic, the free-living setting allowed for additional assessment of sleep and fatigue, which were performed in multiple ways. The PVT test was performed daily and allowed for specific measurement of reaction times.

### Sleep assessments

Participants also tagged when they were sleeping each night; during these periods, data were analyzed for heart rate variability as well as body movement, particularly lower leg motion, which should generally be absent during sleep. Trained models correlating heart rate variability with rapid-eye movement (REM) sleep stages were used to calculate the total % of REM sleep during patient tagged sleep segments.

### Statistical analysis

All continuous variables were presented using the mean and standard deviation, while categorical variables were presented as number of individuals and percentages. Feasibility was assessed by (1) patient visit completion, (2) free-living adherence, and (3) percent assessments completed. To assess the potential correlation between disability measures (EDSS, MSFC-4) and the in-clinic device features, Spearman’s rank correlation coefficients were calculated between each pair of features. Storey’s *q*-values^30^ were used to correct for multiple testing with the *q*-value library in R. All remaining statistical analyses were completed in SAS 9.4.

### Reporting summary

Further information on research design is available in the Nature Research Reporting Summary linked to this article.

## Supplementary information

Supplementary Information

Reporting Summary

## Data Availability

The derived data that support the findings of this study are available from the corresponding authors upon reasonable request.
