# Supplementary material for: Quantifying neurologic disease using biosensor measurements in-clinic and in free-living settings in multiple sclerosis
Source: NPJ Digit Med. 2019 Dec 11;2:123. doi: 10.1038/s41746-019-0197-7 (PMC6906296; doi:10.1038/s41746-019-0197-7)
Supplement: Supplementary file 1 — Supplementary Information [file 41746_2019_197_MOESM1_ESM.pdf]

Supplemental data description of extracted biosensor features used in this study:

Mobility features (used both in-clinic and free-living):
(note: these are calculated both for structured data and walking segments annotated in unstructured data; for the free-living data the median values for the feature calculated for each day were used in the primary analysis.)

Gait

- stance (s) - The number of seconds during the gait cycle during which the foot is on the ground. This is estimated from the ankle-worn gyroscopes.
- swing (s) - The number of seconds during the gait cycle during which the foot is not in contact with the ground (swinging). This is estimated from the ankle-worn gyroscopes.

Turns (note: each of these parameters is calculated independently from data for the chest-worn gyroscope and ankle-worn gyroscopes):

- turn_angle (deg) - Total angle of turn from [ankle/chest]-worn gyroscope, from detected turn events during walking (using the wrist-worn accelerometer if classifying from free-living data).
- turn_duration (s) - The time length of turn events, measured in seconds.
- turn_vel_max (deg/s) - The maximum angular velocity (measured from the [ankle/chest]-worn gyroscope, in degrees/second) during turn events.
- turn_vel_mean (deg/s) - The average angular velocity (measured from the [ankle/chest]-worn gyroscope, in degrees/second) during turn events.
- turn_vel_std (deg/s) - The median over each day of the standard deviation of angular velocity (measured from the [ankle/chest]-worn gyroscope, in degrees/second) during turn events.

Additional in-clinic features from structured activities:
(note: these structured activities were also performed twice a week at home during the free-living section in addition to the unstructured data collection, with the exception of the PVT and ECG tests which were performed daily):
Mobility (gait and balance)

- mobility_activity_time (ms) - Time taken in milliseconds to perform the walking portion of the mobility activity, measured algorithmically from step detection.
- sway_dist_LR (mm) - Left-right distance in millimeters traveled during the postural stability (sway) portion of the mobility activity.
- sway_dist_AP (mm) - Anterior-posterior distance in millimeters traveled during the postural stability (sway) portion of the mobility activity.
- sway_disp_LR (mm) - Left-right displacement in millimeters during the postural stability (sway) portion of the mobility activity.
- sway_disp_AP (mm) - Anterior-posterior displacement in millimeters during the postural stability (sway) portion of the mobility activity

Fatigue

- mean_pvt_delay (ms) - The average over all challenges within one test of the Psychomotor Vigilance Test (PVT) response time (delay from challenge to tap event) in milliseconds.
- fatigue_level - A self-reported fatigue score entered at the time of a PVT test start. The wrist device displays the question “How fatigued are you?” with 5 choices ranging from 1 = “NOT AT ALL” to 5 = “WORST POSSIBLE” that the participant tags on the device.
- mean_pvt_delay_{1,3,5,7} (ms) - The average response time in milliseconds over the first 1, 3, 5, or 7 challenges within a PVT test.

Heart rate

- hrv_sdnn (ms) - Heart rate variability (standard deviation of R-R peak interval) in milliseconds during ECG collected from the wrist-worn devices. Will be NaN when ECG signal quality is too poor to produce a confident result.

Additional free-living only features from unstructured data:

Activity

- walk_minutes (min) - Number of minutes of walking or running time in a day, as detected by the wrist-worn activity classifier.
- idle_minutes (min) - Number of minutes of idle time (including sleep), as detected by the wrist-worn activity classifier.

Pulse rate

- prv_sdnn (ms) - Standard deviation of peak-to-peak intervals, in milliseconds, detected from the pulse rate sensor when the wrist-based activity classifier indicated "idle" activity. Used at night to classify stages of sleep.

Sleep

- movement_rate (count / hr) - The mean number of movement events per hour of sleep, detected at the wrist.
- duration (s) - The length of sleep, as reported manually by the participant based on tagging of the start and end of sleep each night on the wrist-worn biosensor.
- rem_epochs (count) - Count of REM epochs (30-second windows) detected during manually tagged sleep.
- rem_percent (percentage) - Fraction of epochs (30-second windows) during manually tagged sleep that were in REM sleep.

Supplemental Figure 1: Subject compliance during the free-living observation period. Shown is the overall wear time per day for both wrist devices (one meant to be worn during the day and the other at night, but participants could wear both at the same time). The protocol instructed participants to wear for 20 hours (yellow line) and the median wear time observed throughout the study was close to this line.

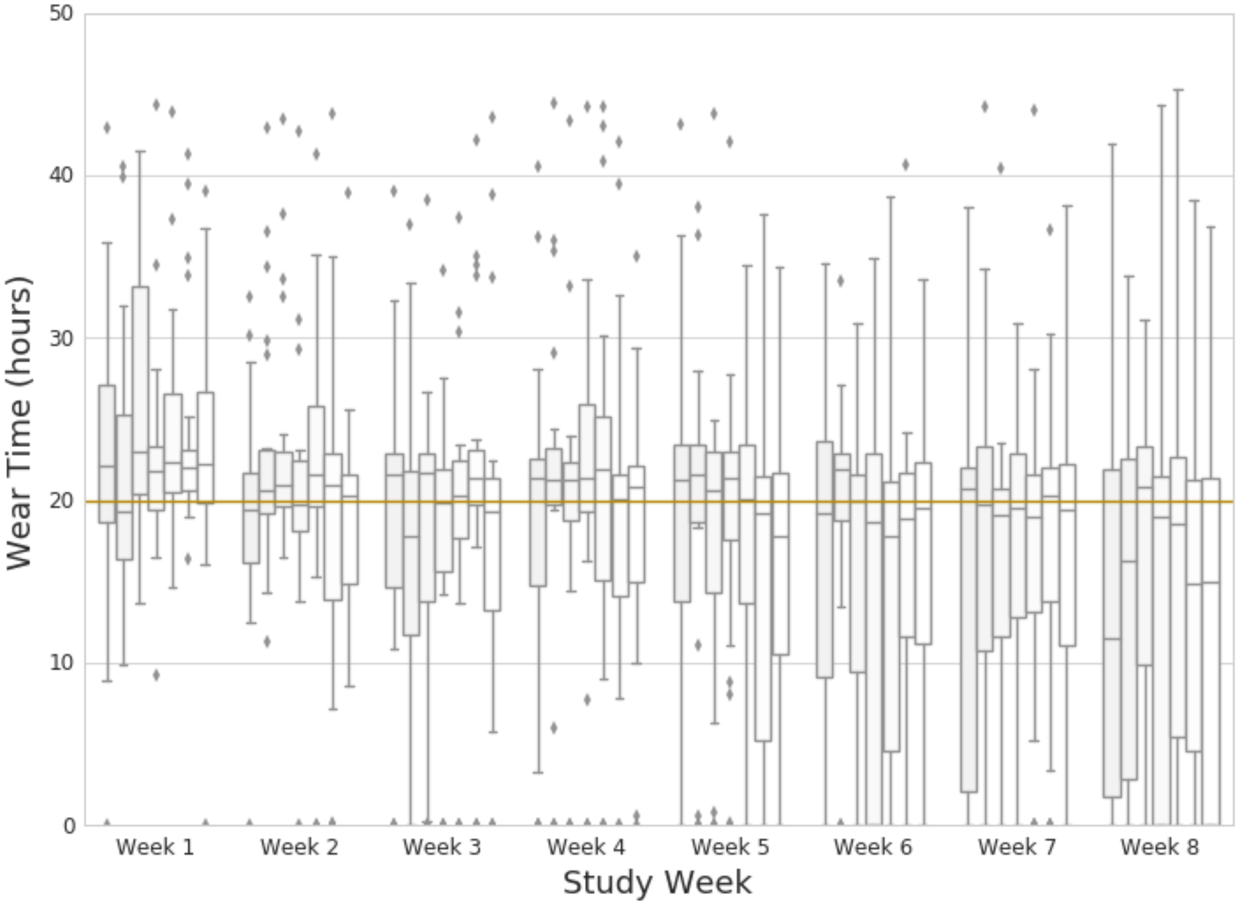

Supplemental Table 1: Study demographics for the 25 enrolled participants.

**Characteristics**	**Total (n = 25 subjects)**
Age (yrs)	46.5 ± 7.4
Height (inches)	64.5 ± 3.3
Weight (lbs)	190.5 ± 51.8
BMI	32.5 ± 10.0
Average MS disease duration (yrs)	16 ± 5
Sex: Female	23 (92%)
Sex: Male	2 (8%)
EDSS at baseline: subjects from 0 to 2.5	11 (44%)
EDSS at baseline: (group: >2.5 to 4.0)	7 (28%)
EDSS at baseline: (group: >4.0 to 6.5)	7 (28%)
Average EDSS score at Visit 1 (25 subjects)	3.38 ± 1.86
Average EDSS score at Visit 2 (23 subjects)	3.46 ± 1.85
Average EDSS score at Visit 3 (21 subjects)	3.43 ± 1.96

Supplemental Table 2: Spearman correlations of 23 in-clinic biosensor and structured testing measures with EDSS, MSFC-4, and MSFC-4 composite z-scores as observed at the first clinic visit.

Category	Feature	N	EDSS	MSFC4 z-score	25 Foot Walk z-score	9 Hole Peg Test z-score	Symbol-Digit Modality Test z-score	LCVA Test z-score
Mobility (gait)	Stance time	24	0.674**	-0.479*	-0.717**	-0.360	-0.076	-0.330
	Swing time	24	0.597**	-0.339	-0.642**	-0.314	-0.105	-0.062
	Mobility activity time	24	0.719**	-0.628**	-0.678**	-0.715**	-0.507*	-0.213
Mobility (turn)	Turn angle - chest	25	-0.492*	0.411*	0.440*	0.355	0.250	0.274
	Turn duration - chest	25	-0.242	0.267	0.258	0.210	0.277	0.189
	Turn velocity (max) - chest	25	-0.544*	0.482*	0.519*	0.392	0.244	0.301
	Turn velocity (mean) - chest	25	-0.511*	0.375	0.417*	0.320	0.196	0.234
	Turn velocity (std) - chest	25	-0.470*	0.645**	0.436*	0.528**	0.415	0.360
	Turn angle – ankle	25	-0.116	0.084	0.136	0.108	0.158	-0.055
	Turn duration - ankle	25	0.041	-0.142	-0.068	-0.191	-0.169	-0.156
	Turn velocity (max) - ankle	25	-0.205	0.188	0.326	0.111	0.215	0.126
	Turn velocity (mean) - ankle	25	-0.008	-0.028	0.052	-0.006	-0.003	-0.073
	Turn velocity (std) - ankle	25	-0.343	0.418*	0.611**	0.319	0.306	0.352
Mobility (balance)	Sway distance left-right	25	0.443*	-0.265	-0.261	-0.399	-0.159	-0.127
	Sway distance anterior-posterior	25	0.452*	-0.258	-0.22	-0.269	-0.046	-0.070
	Sway displacement left-right	25	0.397*	-0.250	-0.333	-0.263	-0.110	-0.090
	Sway displacement anterior-posterior	25	0.293	-0.352	-0.027	-0.340	-0.351	-0.167
Overall activity	Heart rate variability	14	-0.077	0.174	0.220	0.174	0.011	-0.110
Fatigue	Mean PVT delay - Total	25	0.404*	-0.502*	-0.473*	-0.504*	-0.590**	-0.267
	Mean PVT delay - 1 challenge	25	0.436*	-0.651**	-0.494*	-0.694**	-0.705**	-0.350
	Mean PVT delay - 3 challenges	25	0.449*	-0.624**	-0.483*	-0.715**	-0.713**	-0.270
	Mean PVT delay - 5 challenges	25	0.528*	-0.555*	-0.495*	-0.679**	-0.605**	-0.204
	Mean PVT delay - 7 challenges	25	0.521*	-0.535*	-0.504*	-0.676**	-0.609**	-0.164

Legend: EDSS: Expanded disability status scale; MSFC-4: Multiple sclerosis functional composite-4; LCVA: Low contrast visual acuity test; *p- and q-values <0.05; **p- and q-values <0.01.

Supplemental Table 3: Spearman correlations of 23 in-clinic biosensor and structured testing measures with EDSS, MSFC-4, and MSFC-4 composite z-scores as observed at each of the third clinic visits.

Category	Feature	N	EDSS	MSFC4 z-score	25 Foot Walk z-score	9 Hole Peg Test z-score	Symbol-Digit Modality Test z-score	LCVA Test z-score
Mobility (gait)	Stance time	17	0.588	-0.446	-0.615*	-0.336	-0.173	-0.228
	Swing time	17	0.320	-0.017	-0.328	0.184	0.156	-0.047
	Mobility activity time	17	0.193	-0.279	-0.590*	-0.066	-0.307	-0.276
Mobility (turn)	Turn angle - chest	19	-0.210	0.277	0.163	0.495	-0.271	0.314
	Turn duration - chest	19	-0.076	0.162	0.137	0.421	-0.227	0.044
	Turn velocity (max) - chest	19	-0.268	0.328	0.263	0.540	-0.199	0.249
	Turn velocity (mean) - chest	19	-0.147	0.191	0.082	0.374	-0.382	0.291
	Turn velocity (std) - chest	19	-0.223	0.274	0.462	0.361	-0.041	0.185
	Turn angle – ankle	19	0.181	-0.047	-0.226	-0.126	0.051	0.285
	Turn duration - ankle	19	0.408	-0.210	-0.551*	-0.114	-0.068	-0.072
	Turn velocity (max) - ankle	19	-0.263	0.426	0.442	0.325	0.367	0.439
	Turn velocity (mean) - ankle	19	0.272	-0.123	-0.251	-0.214	0.027	0.220
	Turn velocity (std) - ankle	19	-0.340	0.354	0.549*	0.293	0.340	0.129
Mobility (balance)	Sway distance left-right	19	0.480	-0.400	-0.649*	-0.402	-0.155	-0.054
	Sway distance anterior-posterior	19	0.487	-0.337	-0.679*	-0.339	-0.075	0.071
	Sway displacement left-right	19	0.354	-0.263	-0.437	-0.305	-0.061	0.169
	Sway displacement anterior-posterior	19	0.421	-0.237	-0.521*	-0.140	-0.308	0.289
Overall activity	Heart rate variability	13	0.059	-0.225	-0.160	-0.143	0.022	-0.405
Fatigue	Mean PVT delay - Total	21	0.297	-0.405	-0.327	-0.233	-0.541	-0.363
	Mean PVT delay - 1 challenge	21	0.064	-0.260	-0.087	-0.096	-0.367	-0.248
	Mean PVT delay - 3 challenges	21	-0.021	-0.072	-0.071	0.087	-0.299	-0.105
	Mean PVT delay - 5 challenges	21	0.109	-0.125	-0.175	0.069	-0.260	-0.176
	Mean PVT delay - 7 challenges	21	0.173	-0.244	-0.234	-0.042	-0.388	-0.265

Legend: EDSS: Expanded disability status scale; MSFC-4: Multiple sclerosis functional composite-4; LCVA: Low contrast visual acuity test; *p- and q-values <0.05; **p- and q-values <0.01.

Supplemental Table 4: Description of how the MSFC-4 and MSFC-4 composite z-scores were calculated.

Feature	Raw score calculation	Mean and SD	z-score calculation
T25FW	The average of the two walk trials	Calculate mean and SD of the raw T25FW score	-1×((T25FW raw score – mean)/ SD)
9HPT	The average of the two trails for each hand, then calculate the inverse average	Calculate mean and SD of the raw 9HPT score	((9HPT raw score – mean)/ SD)
LCVA	The numerator divided by the denominator for the Snellen score	Calculate mean and SD of the raw LCVA score	((LCVA raw score – mean)/ SD)
SDMT	Use the SDMT score that was generated	Calculate mean and SD of the raw SDMT score	((SDMT raw score – mean)/ SD)
MSFC-4			The average of the 4 calculated composite sub-scores
